# Sphincterochilidae from Tunisia, with a note on the subgenus *Rima* Pallary, 1910 (Gastropoda, Pulmonata)

**DOI:** 10.3897/zookeys.151.2264

**Published:** 2011-12-03

**Authors:** Intidhar Abbes, Said Nouira, Eike Neubert

**Affiliations:** 1 UR Biologie et Ecologie des populations, Institut supérieur des Sciences Biologiques de Tunis, 9 Rue Zouhaier Essafi, 1007 Tunis, Tunisie; 2 Naturhistorisches Museum der Burgergemeinde Bern, Bernastr. 15, CH-3005 Bern, Switzerland

**Keywords:** *Sphincterochila candidissima*, *Sphincterochila tunetana*, *Rima*, taxonomy, anatomy, distribution data, *Sphincterochila candidissima*, *Sphincterochila tunetana*, *Rima*, taxonomie, anatomie, distribution

## Abstract

In order to establish an updated checklist of terrestrial gastropod from Tunisia, a revision of the species of Sphincterochilidae is presented, using bibliographic and museum records and the results of our own field work. As a result, only two species, *Sphincterochila candidissima* and *Sphincterochila tunetana*, are accepted to occur in Tunisia, and their type specimens are illustrated. The study of the morphological characters of the genital organs of both species clarified their subgeneric affiliation. Comparison of *Sphincterochila tunetana* with *Sphincterochila cariosa* from Lebanonshowed that the first has to be classified within the subgenus *Albea*, and the latter within *Sphincterochila* s. str.; the subgenus *Rima* Pallary, 1910 remains in the synonymy of *Sphincterochila* s. str. Bibliographic records of *Sphincterochila baetica* and *Sphincterochila otthiana* from Tunisia could not be confirmed, the latter probably lives close to the border with Algeria.

## Abstract (fr)

Dans le but d’établir une liste actualisée des gastéropodes terrestres de la Tunisie, la révision des espèces appartenant à la famille des Sphincterochilidae, basèe sur les études bibliographiques et muséologiques et sur les résultats de nos propres travaux de terrains, a été présentée. Le résultat obtenu montre que deux espèces uniquement, *Sphincterochila candidissima* et *Sphincterochila tunetana*, sont trouvées en Tunisie, les types de ces espèces ont été illustrés. L’étude des caractères morphologiques des organes génitaux de ces deux espèces a clarifié leur affiliation subgénérique. La comparaison de *Sphincterochila tunetana* avec *Sphincterochila cariosa*, du Liban, montre que la première espèce doit être classifiée au sein du sous genre *Albea* et la seconde sous le genre *Sphincterochila* s. str ; le sous genre *Rima* Pallary, 1910 reste un synonyme de *Sphincterochila* s. str. Le recensement bibliographique de *Sphincterochila baetica* et *Sphincterochila otthiana* de la Tunisie n’a pas pu être confirmé, la dernière pourrait vivre sur la frontière Algérienne

## Introduction

The systematic position of members of this family has been debated since almost 150 years. Bourguignat (1853) followed Moquin-Tandon (1848) by placing species into the genus *Zonites (Calcarina)* s. lat. because of the presence of an oxygnathous mandible. Later, this genus was placed within a broad “Helicidae” assemblage by Albers (1860), although particular characters of the genital organs have been known quite early (for a comprehensive review we refer to Hesse 1931: 98). Westerlund (1886) separated the group under the family name Leucochroidae, thus excluding it from the “Helicidae” sensu auctores (for nomenclatural details refer to Schileyko 2004; Bouchet and Rocroi 2005).

The family Sphincterochilidae is quite specious, its representatives can be found from Morocco throughout northern Africa to Greece, southern Turkey and the Levant area, but also on the Iberian Peninsula, southern France and the southern parts of Italy and Malta. One radiation centre is situated in northwestern Africa, which is inhabited by a variety of conchologically differing shells. In the 19^th^ century, Michaud (1833), Terver (1839), Bourguignat (1864), Letourneux and Bourguignat (1887), Pfeiffer (1850), and Kobelt (1888) added numerous species-level taxa from this area. Later, (Pallary (1910, 1918 etc.) enriched the system with an additional number of names, and finally Llabador (1950). Zilch (1966) listed the type specimens he identified in the collection of the SMF, and Forcart (1972) supplied information on the anatomy of several species and presented a new generic system of the family. Hausdorf (1998) reviewed the position of the family within Helicoidea, and Schileyko (2004) summarised the system (merely the same system as proposed by Forcart with the exception of retaining *Rima* Pallary, 1910 as a subgenus). At the species level, approximately 100 nomenclaturally available names exist, but a revision is pending leaving a quite unclear and unsatisfactory situation as far as the actual number of species/subspecies is concerned.

This article mainly focuses upon the species occurring in Tunisia, but some problematic taxa from neighbouring countries are addressed as well. Letourneux and Bourguignat (1887) as the first (and only) comprehensive source on the malacofauna of Tunisia reported three species from Tunisia, i.e. *Leucochroa candidissima*, *Leucochroa baetica* and *Leucochroa otthiana* including several varietal forms, and misinterpreted *Helix tunetana* Pfeiffer 1850 as a hygromiid. We here describe morphological details of the shell and characters of the genital organs in order to add to the knowledge of these species. Additionally, type specimens are illustrated to support our identifications and to facilitate further work on the species-level taxa. As *Sphincterochila tunetana* was conchologically allocated by Pallary (1910) to the subgenus *Rima* Pallary, 1910, the status of this subgenus is shortly reviewed.

## Material and methods

Specimens were collected by hand during field studies conducted in Tunisia since 2005. Living animals were drowned in water for 36 to 48 hours and then fixed in 75% ethanol. Animals were dissected under a stereomicroscope using thin pointed watchmakers’ forceps. Anatomical details were drawn using a Wild camera lucida or photographed. Geographic coordinates of the sampling stations were recorded using a GPS, and a map illustrating the distribution of the species found to live in Tunisia is provided. All shell figures are scaled × 3 to provide a comparative aspect.

Key to acronyms used in figures: A – genital atrium; Ag – albumen gland; Bc – bursa copulatrix; Dbc – duct of bursa copulatrix; Div – coecum-like diverticulum; E – epiphallus; Osd – ovispermiduct; Pc – penial coecum; Ped – pedunculus; Mrp musculus retractor penis; Sg – stimulator gland; Sta – stimulator appendix; Std – stimulator duct; V – vagina; Vd – vas deferens.

Acronyms of collections studied: MHNG – Musèum d’Histoire Naturelle Genève; NEUB – private collection E. Neubert, Badenweiler; NHMW – Naturhistorisches Museum Wien; NMBE – Naturhistorisches Museum der Burgergemeinde Bern; SMF – Naturmuseum Senckenberg, Frankfurt.

## Systematics

### Sphincterochila ( Albea ) candidissima

(Draparnaud, 1801)

http://species-id.net/wiki/Sphincterochila_candidissima

Figs 1 –4 5A

Helix candidissima Draparnaud, 1801: 75.
Zonites candidissimus , – Bourguignat 1863: 85.
Zonites candidissimus maxima Bourguignat, 1863: 87.
Zonites candidissimus , – Bourguignat 1864: 322.
Zonites candidissima , – Bourguignat 1868: 10.
Leucochroa candidissima , – Issel 1885: 6.
Leucochroa candidissima , – Letourneux and Bourguignat 1887: 3.
Leucochroa baetica , – Letourneux and Bourguignat 1887: 4 [non *Helix baetica* Rossmässler, 1839].
Leucochroa baetica var. tunetana Letourneux & Bourguginat, 1887: 4 [Guelaat es Snam; secondary homonym of *Helix tunetana* L. Pfeiffer, 1850].
Leucochroa candidissima , – Ktari and Rezig 1976: 37.

#### Type specimens

*candidissima*: syntype NHMW 14810, D = 17.95 mm [no original label left, type locality: France, «en Provence et dans le Comtat»; *maxima*: not identifiable in MHNG; *tunetana*: MHNG 3896, D= 20.0 mm.

#### Material examined

**Bizerte**: Aïn Ezzommita, N 36.87628, E 9.64936, 23.12.08, coll. Abbes/8; Barrage El Khadhra, N 36.16681, E 10.06214, 14.12.08, coll. Abbes/4; Barrage El Khirba, N 37.16354, E 10.0955, 21.02.08, coll. Abbes/6; Utique, N 37.04007, E 1003244, 02.03.05, coll. Abbes/9; **Nabeul**: NEUB 02879, Wadi NE of the city, 36°28'N 10°45'50"E, 26.-31.12.1993, leg. B. & R. Kinzelbach; **Ariana**, Djebel Bejewa, N 37.03027, E 10.027040, 11.04.08, coll. Abbes/12; Ichkeul National Park, N 37. 11255, E 9.34953, 04.03.07, coll. Abbes/7; **Tunis**: NEUB 02878, Tunis, 20 km S of Hamamet, 02.03.1993, leg. J. Gugel; **Ben Arous**: Djebel Boukornine, N 36.4122, E 10.2125, 4.01.07, coll. Abbes/8; Djebel Reças, N 36.59382, E 10.3194, 09.10.08, coll. Abbes/5; **Nabeul**: Assomaa, N 36.52552, E 10.77991, 04.02.09, coll. Abbes/3; Korbos, N 36.82950, E 10.57071, 03.02.09, coll. Abbes/8; **Zaghouan**: Djebel Zaghouan, N 36.37543, E 10.11868, August 2008, coll. Abbes/9; Djebel Zriba, N 36.37, E 10.11, 20.08.08, coll. Abbes/1; **Beja**, Nefza, N 97.0041, E 9.08434, 4.4.05, coll. Abbes/2; **Jendouba**, Ouechteta, N 36.96445, E 9.01706, 24.12.08, coll. Abbes/4; **El Kef**: Djebel Boujeber, N 35.73791, E 8.27292, 26.12.08, coll. Abbes/3; **Sousse**, Tekrouna, N 36,085749, E 10,182551, 25.08.2008, coll. Abbes/2; **Siliana**: Ain tejra, N 36.26, E 9.43, 07.11.08, coll. Abbes/16; **Kairouan:** Aïn Chrichira, N 35.63908, E 9.80950, 14.12.08, coll. Abbes/9; Sbikha, N 35.98503, E 10.03106, 14.12.08, coll. Abbes/17; Djebel Serj, N 36.04555, E 9.63311, 28.12.08, coll. Abbes/2; **Mehdia,** Dowwira, N 35.26905, E 11.09773, 19.03.09, coll. Abbes/4; **Sidi Bouzid**: Bouhedma National Park, N 34.185722, E 245520, 11.05.08, coll. Abbes/10; NEUB 02875, Jebel Bou Hedma, 15-18.03.1993, leg. J. Gugel; NEUB 02876, Sabkhat Mecheguig, ca. 50 km S Kairouan, 02.03.1993, leg. J. Gugel; **Kasserine:** Djebel Chaambi, N 35.10139, E 8.40486, 30.11.08, coll. Abbes/8; Table de Jugurtha, 20.05.09, coll. Abbes/11; **Kebili:** NEUB 02874, big erg close to Ksar Rhilane, 32°59'N 9°38'E, 09.03.1993, leg. J. Gugel; NEUB 02880, Kebili, 30 km S of the city, 33°29'N, 9°02'E, 12.03.1993, leg. J. Gugel; **Tataouine:** NEUB 02877, Tataouine, 32°56'N, 10°27'E, 05.03.1993, leg. J. Gugel; NEUB 02881, Gouvernorat de Tataouine, Ramadah, 32°19'N, 10°24'E, 05.03.1993, leg. J. Gugel.

#### Diagnosis

Shell medium sized, helicoid, globose, shell walls thick, external surface slightly wrinkled, last whorl rounded, aperture rounded, umbilicus closed.

#### Description

(Figs 2–4). Shell medium sized, helicoid, globose, spire slightly depressed; shell colour white; protoconch consisting of two smooth whorls; shell walls thick, teleoconch of six nearly flattened whorls, last whorl large, rounded and sometimes inconspicuously keeled and slightly descending below the periphery of the shell; suture shallow to moderately deep; upper teleoconch surface smooth or with fine irregularly shaped wrinkles; aperture dorsoventrally depressed; peristome discontinuous, only slightly thickened; umbilicus closed by a thick reflection of the columellar peristome. — Measurement (n = 20). H = 17.3 mm ± 2.46; D = 20.57 mm ± 1.33.

#### Anatomy of genital organs

(Fig. 5A). Penis thick with a short and blunt penial coecum, epiphallus a long and cylindrical slender tube reaching 4 × the length of the penis, penial papilla missing; flagellum relatively long; Mrp inserts at the distal third of the epiphallus.

Stimulator gland large, stimulator appendix branches off in a basal position; stimulator duct short, pointing into the large genital atrium with a small papilla.

Vagina very short and slender; pedunculus reaching half of the lenght of the whole bursa copulatric complex, diverticulum short and thickened, bursa copulatrix a well rounded vesicle.

#### Geographic range

**Figure 1.**
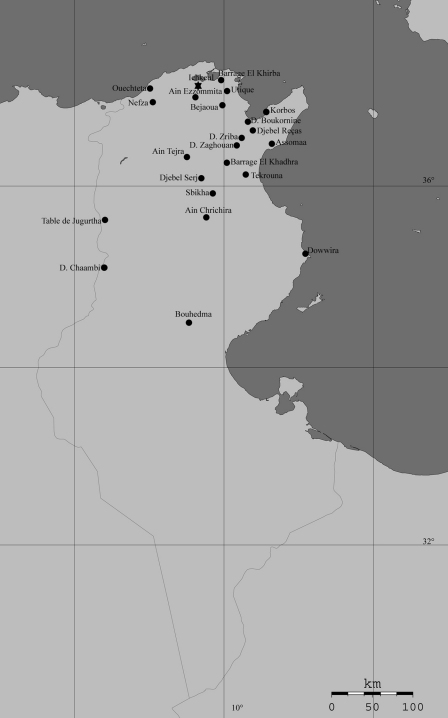
Distribution of *Sphincterochila* species in Tunisia ● *Sphincterochila candidissima* (Draparnaud, 1801) ★ *Sphincterochila tunetana* (Pfeiffer, 1850).

This is a species of western Mediterranean distribution (Giusti et al. 1995), but its actual presence in NW Africa has to be corroborated by a serious investigation of all specimens available including dissections of preserved specimens. Its hitherto known distribution in Tunisia is given in Fig. 1.

#### Remarks

The species *Sphincterochila baetica* (Rossmässler, 1839), which was described from Spain (between Almeria and Venta del Pobre) has been reported for Northwestern Africa from Morocco to Tunisia (Bourguignat 1863; Letourneux and Bourguignat 1887; Morlet 1881; Rour et al. 2002). We here figure one syntype of *Helix baetica* (SMF 7669, Fig. 6) to show the differences between the two species: next to the size difference, shells of *Sphincterochila candidissima* are usually smooth or show a fine sculpture of small wrinkles, while *Sphincterochila baetica* has much stronger and coarse wrinkles or is even malleated, particularly on the upper whorls; in addition, shells of *Sphincterochila candidissima* ususally have a rounded periphery of the last whorl (exceptions see our Figs 3 and 4), while *Sphincterochila baetica* shows a bluntly angulated last whorl. The shell of *Leucochroa baetica* var. *tunetana* might mislead to an identification as *Sphincterochila baetica* as it is quite large and shows the angulation. However, it has the typical finely striated surface sculpture of *Sphincterochila candidissima*, and thus can be synonymised with this species. The collection of Bourguignat in Geneva did not contain any specimen from northwestern Africa that could positively be identified with *Sphincterochila baetica*. However, his collection is particularly weak concerning shells from Morocco, so we are not able at the moment to judge about records from Algeria or Morocco. So far, all specimens we have seen from Tunisia can be identified with *Sphincterochila candidissima*.

The morphological details of the genital organs of dissected specimens from Tunisia compare very well with those presented by Forcart (1972: figs 8, 9) from southern France and Giusti et al. (1995) from Malta.

**Figures 2–4.**
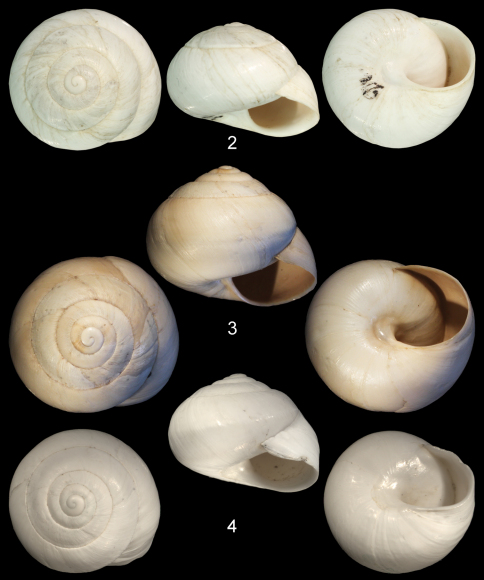
*Sphincterochila candidissima* (Draparnaud, 1801) **2** syntype ex NMHW, France, probably from the Provence **3** syntype of *Leucochroa baetica var. tunetana* Letourneux & Bourguginat, 1887, MHNG 3896, Tunisia, Guelaat es Snam **4** NMBE, Ain Tejra, 07.11.2008, leg. I. Abbes.

**Figure 5.**
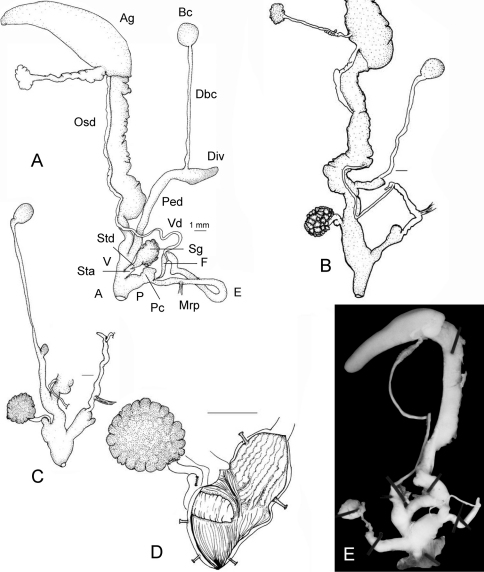
Anatomical details. **A** *Sphincterochila candidissima*, situs of genital organs. **B–D** *Sphincterochila tunetana*. **B** **C** situs of genital organs of two specimens **D** Detail stimulator gland showing the large stimulator papilla pointing into the atrial lumen. – Figs A–C at the same scale. Figure 5E. *Sphincterochila cariosa* (Olivier, 1801). Situs of genital organs (shell illustrated in Fig. 10); length of complete situs 14.9 mm.

### Sphincterochila ( Albea ) tunetana

(Pfeiffer, 1850)

http://species-id.net/wiki/Sphincterochila_tunetana

Figs 1 5B–D 7, 8

Helix tunetana Pfeiffer, 1850: 70 [Habitat circa Tunis Africae].
Helix tunetana , – Pfeiffer 1853: 346-347, pl. 134, fig. 3–4 [syntype figured].
Helix tunetana , – Reeve 1854: pl. CXCIX fig. 1400.
Helix tunetana , – Letourneux and Bourguginat 1887: 94.
Albea tunetana , – Pallary 1939: 67.

#### Type specimens

No type specimens could be traced in any larger museum collection with holdings of specimens from the Pfeiffer collection. One syntype was figured by Pfeiffer in 1853 (“in der Gegend von Tunis, aus H. Cuning’s Sammlung”), another without reference to the collector by Reeve in 1854.

#### Material examined

Ichkeul National Park 13.02.08, coll. Abbes/15.

#### Diagnosis

Shell nearly flat or with slightly elevated spire, upper shell surface with very coarse sculpture; last whorl keeled, aperture lenticular; umbilicus open to completely closed.

#### Description

Shell medium sized, nearly flat or with slightly elevated spire; shell with 4 ½ flattened and regularly growing whorls; white yellowish in colour; suture shallow; upper shell surface with coarse and oblique, rib-like sculpture, lower shell surface with irregular wrinkles; last whorl sharply keeled; aperture lenticular; peristome discontinuous, parietal callus lacking; lip slightly thickened, often slightly reflected on the lower and columellar side; umbilicus wide and open, surrounded by a cord like ridge; there are specimens where the columellar reflection completely obscures the umbilicus.

#### Measurement.

(n = 15). H = 8.5 mm ± 1.37; D = 16.58 mm ± 0.79.

#### Anatomy of genital organs

(Figs 5B–D). Penis thick club-shaped, with a short penial coecum, epiphallus long reaching only twice the length of the penis, penial papilla missing; flagellum short; musculus retrator penis inserts at the distal third of the epiphallus.

Stimulator gland very large, stimulator appendix branches off in a basal position; stimulator duct short, pointing into the genital atrium with a large papilla (Fig. 5D).

Vagina very short and slender; pedunculus short, reaching a third or even less of the lenght of the whole bursa copulatric complex, diverticulum short to reduced, bursa copulatrix a well rounded vesicle.

#### Distribution

This species was only reported from Tunisia by Letourneux and Bourguignat (1887) and from Algeria by Bourguignat (1864).

#### Remarks

At first glance, *Sphincterochila tunetana* may be confused with a species of the Hygromiidae, *Helicopsis (Xeroleuca) degenerans* Mousson, 1872, from Morrocco (Fig. 9) because of the depressed shape of the shell, the magnificent sculpture, and the open umbilicus. However, the small-sized protoconch of *Helicopsis degenerans* is a good character to discriminate it from *Sphincterochila tunetana*. Affiliation of the latter species to the Sphincterochilidae was already suggested by Pallary (1901, 1910).

The differences in morphology of the genital organs to *Sphincterochila candidissima* are quite large: *Sphincterochila candidissima* has a much longer epiphallus and flagellum, and the diverticulum in *Sphincterochila tunetana* seems to be reduced, and its pedunculus is considerably shorter than in *Sphincterochila candidissima*.

**Figure 6.**
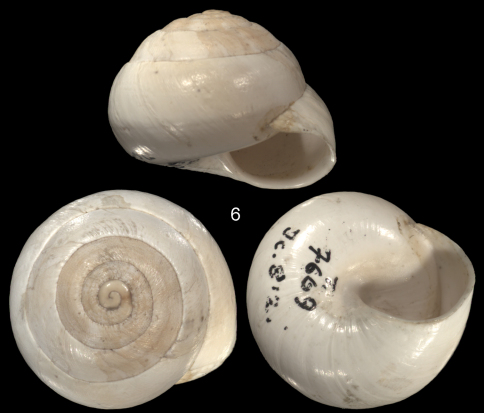
*Sphincterochila baetica* (Rossmässler, 1839), syntype SMF 7669, Spain, between Almeria and Venta del Pobre, coll. Rossmässler (= Orig. Icon. 812).

**Figures 7–10.**
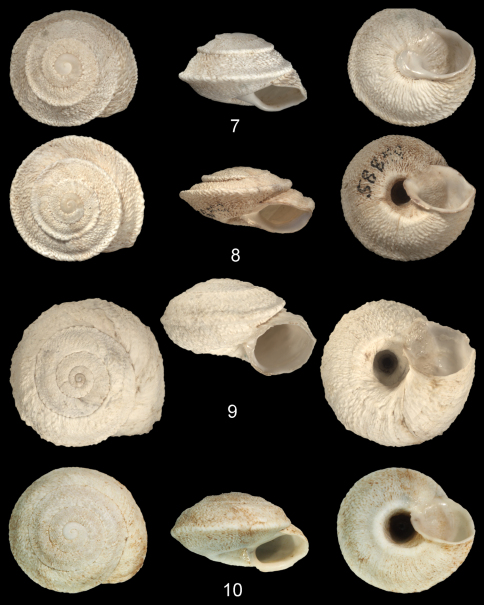
7, *Sphincterochila tunetana* (Pfeiffer, 1850), NMBE, Tunisia, Ischkeul, 08.01.2004, leg. I. Abbes **8** *Sphincterochila tunetana* (Pfeiffer, 1850), SMF 58859, Achkeul [= Ischkeul], ex coll. Pallary **9** *Xeroleuca degenerans* (Mousson, 1876), syntype ZMZ 502781, Morocco, “Ain Umest westlich der Maroccoebenen, coll. Mousson ex Fritsch 1873” **10** *Sphincterochila cariosa* (Olivier, 1801), Libanon, Deir el Kamar, 33.7°N 35.59°E, leg. N. Sayar, 4.10.2009 (shell of anatomically investigated specimen).

## Discussion

**On the subgeneric classification**

The history of the subgeneric classification of *Sphincterochila* Ancey, 1887 was demonstrated by Forcart (1972: 159). He finally came to the conclusion that there are three subgenera, i.e. *Sphincterochila* s. str. (based on *Helix boissieri* Charpentier, 1847) comprising the Levant radiation of the family, then *Albea* Pallary, 1909 (nom. nov. pro *Calcarina* Moquin-Tandon, 1848, based on *Helix candidissima* Draparnaud, 1801) comprising the African and European taxa, and *Zilchena* Forcart, 1972 (based on *Helix piestia* Bourguignat, 1859) for this single species. All other existing genus-level taxa were synonymised by him under one of these three subgenera. In 1993, Gittenberger added the subgenus *Cerigottella* based on *Leucochroa candidissima* var. *insularis* O. Boettger, 1894, the only *Sphincterochila* species known from this country. He added two species from Libya to this new subgenus. This system was adopted by Schileyko (2004) with the exception of a re-establishment of the subgenera *Rima* Pallary, 1910 (based on *Helix cariosa* Olivier, 1801), and *Cariosula* Pallary, 1910 (based on *Helix cariosula* Michaud, 1833). Both resurrections are not discussed, autapomorphic characters for these two groups were not presented.

Already Pallary (1939) suggested a close relationship between *Sphincterochila tunetana* and *Sphincterochila cariosa* because of the resemblance in shell characters of both species. Based on the anatomy of the genital organs, as published by Schmidt (1855) and Hesse (1931), Forcart (1972) placed *Rima* into the synonymy of *Sphincterochila* s. str. Its resurrection by Schileyko (2004) probably followed the same conchological feature of an open umbilicus in *Sphincterochila cariosa* as already favoured by Pallary (1939).

Forcart (1972) introduced a new approach to the subgeneric classification using particular characteristics of the stimulator organ. As he explained, this organ has a strong-walled distal “sheath” connecting the stimulator to the atrium (condition in *Sphincterochila* s. str.), which is thin-walled in *Albea* and *Zilchena* (the latter has a second stimulator appendix, a character which urgently needs corroboration!).

According to Schileyko’s classification of 2004, the lenticular shape of the shell and the open umbilicus automatically qualifies *Sphincterochila tunetana* for inclusion into the subgenus *Rima*. For this reason, we dissected a specimen of *Sphincterochila cariosa* from the vicinity of Beirut (Lebanon) to compare the morphology of its genital organs to that of *Sphincterochila tunetana*. Our investigation clearly showed that *Sphincterochila tunetana* has a thin-walled “sheath” (Fig. 5B-D), while in *Sphincterochila cariosa* it is clearly thickened (Fig. 5E). As a result, *Sphincterochila tunetana* has to be classified within *Albea*, while *Sphincterochila cariosa* stays in *Sphincterochila* s. str. as suggested by Forcart. However, his classification suffers from the fact that he could not cover the whole radiation of the family, hence his system requires a serious reconsideration. The use of *Cariosula* as a separate subgenus could not be addressed here because of lack of specimens (the species does not occur in Tunisia) and is left for a comprehensive taxonomic revision of the family.

**On records of other nominal taxa of Sphincterochilidae from Tunisia**

Another species recorded by Letourneux and Bourguignat (1887) was *Sphincterochila otthianus* (Forbes, 1839) (type specimen not in NHM London, nor in Edinburgh). The authors recorded it from Cap Roux [= Ras Saklab] close to Tabarka in the NW of Tunisia; no corresponding shells could be found in the collection of Bourguignat at MHNG. This species is characterized by a large white and flattened shell with a conspicuously ribbed surface, a shallow suture, a keeled the last whorl, a discontinuous peristome and a close to slightly open umbilicus. It has to be stressed that Ras Maklab belongs today politically to Algeria and could not be visited by us until now. However, the Tunisian part of the border area was intensively searched for *Sphincterochila*, but no specimens of any species were found so far. Thus, a positive record for *Sphincterochila otthiana* (which is widespread in Algeria) from Tunisia is still missing.

*Sphincterochila candidissima* is one of the most widespread species of the family and was most likely introduced to France. However, it is frequently recorded from NW Africa to Spain, and this probably represents the natural distribution area of the species. Within this range, shell morphological variation may be found as can be seen in southern Tunisia, where the population of Djebel Bouhedma includes specimens with higher and more conical shells if compared to populations from northern Tunisia. By contrast, *Sphincterochila tunetana* has a very restricted geographic range and seems to be endemic to the Ichkeul National Park region.

Many nominal sphincterochilid species from NW Africa have to be reconsidered. For example, S. *maroccana* (Pallary, 1910) looks very similar to *Sphincterochila candidissima*, while some nominal species like *Sphincterochila cariosula* (Michaud, 1833), *Sphincterochila octinella* (Pechaud, 1883), *Sphincterochila rugosa* (Pallary, 1900), and *Sphincterochila corrugata* (Pallary, 1917) show a superficial shell resemblance with *Sphincterochila tunetana*. But as shown in this paper, shell resemblance does not necessarily reflect a phylogenetic relationship, and more basic revisional work is required to entangle the taxonomy and nomenclature of this family as a whole.

## Supplementary Material

XML Treatment for Sphincterochila ( Albea ) candidissima

XML Treatment for Sphincterochila ( Albea ) tunetana
